# Recommending Physical Activity to Your Aging Patients? What Clinicians Need to Know to Increase Adherence From the Older Adult Perspective

**DOI:** 10.3389/fresc.2022.923221

**Published:** 2022-07-08

**Authors:** Danylo F. Cabral, Vinicius S. Santos, Maria Jasmine G. Silva, Gabriela F. Leite, Ana Paula B. Mesquita, Alvaro Pascual-Leone, Lawrence P. Cahalin, Adriane P. Batiston, Augusto C. A. Oliveira, Joyce Gomes-Osman

**Affiliations:** ^1^Department of Physical Therapy, University of Miami Miller School of Medicine, Miami, FL, United States; ^2^Research Group on Human Aging, Department of Physical Therapy, Alagoas State University of Health Sciences, Maceió, Brazil; ^3^Department of Physical Therapy, Federal University of São Carlos, São Carlos, Brazil; ^4^Guttmann Brain Health Institute, Institut Universitari de Neurorehabilitació, Barcelona, Spain; ^5^Hinda and Arthur Marcus Institute for Aging Research and Deanna and Sidney Wolk Center for Memory Health, Hebrew SeniorLife, Boston, MA, United States; ^6^Department of Neurology, Harvard Medical School, Boston, MA, United States; ^7^Linus Health, Waltham, MA, United States; ^8^Federal University of Mato Grosso Do Sul, Campo Grande, Brazil; ^9^Department of Neurology, University of Miami Miller School of Medicine, Miami, FL, United States

**Keywords:** exercise engagement, physical activity, sedentary behavior, exercise guidelines, lifestyle behavior, behavior change, cognitive decline, brain health

## Abstract

A wealth of scientific evidence supports that being physically active may prevent or delay the onset of cognitive impairment and dementia. However, a critical barrier is that while most clinicians recommend physical activity (PA) and older adults recognize its health benefits, most older adults fail to regularly practice PA. Thus, it is necessary to explore and disseminate knowledge on how to help clinicians truly partner with people and help them to change their behavior and become more active. Clinical and scientific efforts are underway to establish dose-specific PA recommendations for cognitive brain health. However, an important knowledge gap is how to develop effective strategies to increase PA adherence in aging. To better understand the perspective of older adults, we undertook a mixed-method study on sixty-five sedentary older adults at risk for cognitive decline. Participants answered a questionnaire battery related to PA engagement, and a subcohort participated in a remote focus group. Our findings revealed four main themes: First, age and aging are determinants in PA practice. Second, maintaining both an active mind and autonomy are priorities, but planned PA is not usually related as part of being “active.” Third, motivational challenges in PA engagement were noted. And fourth, they emphasized a call for tailored recommendations. Therefore, we present a multidimensional model of PA adherence to maximize brain health in older adults and suggest a tool kit and key questions to effectively screen sedentary aging adults and translate current guidelines into the needs of the individual by using behavior change strategies.

## Introduction

### Physical Activity and Sedentary Behavior Change Play a Crucial Role in Promoting Brain Health

Despite the important increase in human longevity (1), living longer (lifespan) has not been followed by an equal or similar increase in living healthy (health-span). As the population ages, many will develop cognitive decline and dementia, leading to substantial loss of health and quality of life (2, 3). Worldwide, there are 55 million individuals over 60 years old living with some form of dementia, and this number is estimated to nearly triple only in the next 30 years, by 2050 (4). *While age is the strongest risk factor for developing cognitive decline, dementia is not an inevitable consequence of aging*. Seemingly simple behaviors such as controlling hypertension and diabetes, increasing physical activity (PA), controlling one's weight, decreasing alcohol consumption, nurturing meaningful social connections, among others, can prevent or reduce the chances of progression to dementia by as much as 40% (5–8). Globally, dementia represented an annual global cost above 1.3 trillion dollars in 2019 and an expected increase up to 3 trillion by 2030 (9, 10).

Being physically active can not only delay but also mitigate the effects of cognitive impairment and dementia (5). Exercising regularly can independently revert age-related hippocampal shrinkage, and actually increase its volume by 2%−4.2%, which promotes improvement in spatial memory function (11, 12). Engaging in regular PA practice can reduce the risk of Alzheimer's Disease by 50% (13, 14). The main physiological mechanisms are linked to increased brain perfusion, synaptic neuroplasticity, increased brain volume and connectivity, and the regulation of neurotrophic factors (15–17). It is critical to appreciate that PA is a modifiable lifestyle factor, and there may be many additional reasons why this is a particularly powerful strategy to promote a healthier lifestyle in aging adults: (1) sedentary behavior is a modifiable lifestyle factor, (2) being physically active can improve important aspects of life such as functional independence, psychological wellbeing, and increase health-span (18–20), and (3) increased PA practice is a powerful cost-effective intervention to prevent and treat chronic diseases (21).

## Progress Made And Remaining Challenges

### Recommending Existing Universal Guidelines for Physical Activity

Numerous organizations have independently endorsed physical activity as a brain-healthy behavior for the aging population (22–26). According to the World Health Organization, a minimum of 150 min of moderate to vigorous activity per week is required for an individual to be considered active (16). Most recently, the PA Guidelines for Americans were updated and mentioned the importance of exercise for brain health and emphasized generally *moving more and sitting less* (22). Taking more daily steps (up to ~8,000 steps) is associated with a progressively lower risk of all-cause of mortality among adults aged 60 years and older (27).

Most healthcare providers recommend that patients increase PA levels (25, 28–31). However, this approach is unfortunately not particularly effective in helping patients to increase their PA levels over a long-term period (32). These recommendations, including step count prescription, are also associated with important limitations as it usually does not consider the singularities (including values and preferences) and environmental context of each individual. There are also limitations in achieving at least moderate intensity exercise when only step counts or a simple 'move more and sit less' are considered. Older adults spend on average 8.5 h daily on sedentary behaviors (33), and less than a third of adults accurately know and understand the PA recommended guidelines (34–37). Worldwide, approximately one-quarter (one-third in women) of this population is physically inactive, and in some countries, the pandemic increased the proportion of physically inactive up to 80% of the population (38, 39).

### Lifestyle Behavior Change Models

Over the last decades, there have been great advances in the science of behavior change. This field has been focused on better understanding the process of behavior change (40). The transtheoretical model (TTM) has been developed focusing on the fact that behavior change is not a discrete task with a beginning middle and end, but a continuous and cyclical process, and is highly dependent on individuals' stage and readiness to change (41). This model can greatly help clinicians as a guide during their conversations about lifestyle change with individuals to gain a better understanding of their specific values, attitudes and needs, and readiness to change, a construct that can be measured with validated instruments (42, 43).

Attempts at appealing to older adults to PA practice have mainly been focused on “selling” the general physical health benefits that one can theoretically gain from becoming physically active. There are several other personal, behavioral, and environmental factors that have been more recently demonstrated to influence exercise adherence in older populations. Social determinants of health have an important impact on one's health, wellbeing, and quality of life (44, 45). Cognitive status and mood can give insights into one's attitudes toward exercise (46). A better understanding of motivation can empower individuals to more successfully pursue their goals (47, 48). The understanding of barriers to exercise and one's self-efficacy is also very important to helping the individuals realize the cyclic nature of behavior change, and anticipate challenges and obstacles (21, 49). Furthermore, self-regulation defined as the planning of how to address barriers can explain exercise engagement (49–51). Finally, a greater understanding surrounding exercise knowledge, tolerance, and preference are critical for setting the correct expectations from the start (52, 53).

Lifestyle interventions that incorporate the concepts discussed above have been successfully employed in helping older adults to promote PA (54), albeit in research settings. In addition, it is relevant to mention motivational interviewing (MI), a patient-centered behavioral approach that focuses on the language associated with behavior change and has been successfully applied to various settings, including increasing PA in older adults (55, 56). However, MI requires specialized training, and time, and may or may not be applicable to most clinical settings. Translation efforts are necessary to develop frameworks that incorporate these theoretical models and adapt them in a way that they can seamlessly be integrated into current workflows of clinical practice.

Thus, to better understand the perspective of the older adult population on PA engagement and adherence and provide supporting data to the proposed perspective, we designed a mixed-method study to explore the main behavioral, biological, social, and environmental factors that affect adherence to regular PA, as well as the PA perception in a sample of sedentary older adults at risk of cognitive decline. Based on this new proposed perspective and results from the mixed-method study, we aim to contrast the current model of PA prescription based mainly on the “universal guidelines,” and the new proposed multidimensional model, which focuses on implementing simple ways to effectively translate current guidelines tailored to the needs of aging individual by screening all listed factors believed to influence adherence and combining all available strategies including health education, evidence-based practice, wellness and lifestyle behavior change models, and behavioral economics and decision-making strategies. We discuss and frame our findings with practical considerations that can hopefully guide clinicians to use the science of behavior change and the concepts to improve their partnerships with their older patients who wish to pursue more active lifestyles.

### Brief Methods

In this perspective study, we support our proposed multidimensional model by collecting data from a mixed-method study (1) a descriptive-exploratory analysis of a structured electronic questionnaires battery related to exercise engagement, and (2) a qualitative content analysis of the focus groups. Participants completed a battery of 11 questionnaires related to demographic characteristics and exercise engagement on the *Qualtrics* platform (Qualtrics, Provo, UT) (Table 1). A total of 65 (67.6 ± 5.9 years, 73% female, 49% brown race, 59% overweighed or obese, 26% high degree, 55% low-to-middle income, 72% retired, 51% hypertension) sedentary older adults (<150 min of moderate or <75 min of vigorous aerobic activity per week) completed the study.

**Table 1:** Toolkit summary and key components for a quick assessment of physical activity adherence-related factors.

**Factors/constructs**	**Definition**	**Outcomes**	**Suggested questionnaires**	**Suggested questions and items to be explored in a quick screening**
1. Sociodemographic characteristics and social determinants of health	All individual biological characteristics and all conditions in the environments that impact people's brain health, wellbeing, and quality of life including birthplace, places where people's live, learn, work, play, worship, and age. The social determinants of health are categorized in 7 domains: general physical health, nutrition, sleep, PA, cognitive activity, socialization, and life plan.	Age, gender, race/ethnicity, body mass index, education, marital status, Household income, occupation, and diseases and morbidities	Demographic form Anamnesis and social determinants of health (45) PA Readiness Questionnaire for Everyone (PAR-Q+) (57)	1. Collect all sociodemographic, medical information, family history, nutrition patterns, social habits 2. How would you describe your present state of health? 3. When was the last time you visited a physician or other health care provider? 4. Do you have any contraindication or precaution to regular PA?
2. Physical and cognitive status	PA level: estimate person's daily physical activities (occupational, leisure-time, and other activities), and is used to estimate daily energy expenditure. Cognitive status: evaluates an individual's state of mind that involves process of learning and understanding through experiences.	PA level Global cognitive status (cognitive reserve and cognitive complaints)	PA Lifetime history questionnaire (58) International PA questionnaire – Short form (59) Montreal cognitive assessment (60)	1. What are your past experiences in practicing regular PA? 2. Do you currently participate in any structured regular PA? 3. Do you carry out cognitively stimulating activities daily? Which activities? 4. Do you feel your memory and thinking is worse than the memory of other people at your age?
3. Mood state	Assess the presence of depressive symptoms and the individual's general perception of stress in relation to their last month	Depressive severity symptoms	Patient Health Questionnaire-9 (PHQ-9) (61)	In the past 2 weeks, how often have you been bothered by… 1. …little interest or pleasure in doing things? 2. …feeling down, depressed, or hopeless? 3. …feeling tired or having little energy? 4. …trouble falling or staying asleep, or sleeping too much? 5.... trouble concentrating on things?
		Perceived stress	Perceived stress scale (PSS) (62)	In the last month, how often… 1. …have you been upset because of something that happened unexpectedly? 2. …have you felt confident about your ability to handle your personal problems? 3. …have you found that you could not cope with all things that you had to do?
3. PA and exercise barriers	Assess participants agreement of a list of most common statements about barriers to PA and exercise.	Perceived barriers	Barriers questionnaire to PA practice in the Elderly (QBPAFI) (63, 64)	In the last 6 months, how often [any item of the below list] get in the way or prevent you from doing physical activities. 1. Lack of company 2. I've being lazy or unmotivated 3. Need of resting and relaxing 4. Running low in money 5. Running out of time
4. Behavior change	Determines the current stage of change proposed by the transtheoretical model.	Stages of change: pre-contemplation, contemplation, preparation, action, maintenance	Stages of behavior change (65)	1. Do you intend to start doing regular PA in the next 6 months? In the next 30 days?
	Assess a person's present motivational state relative to changing a specific behavior	Readiness to change behavior	Readiness to change questionnaire (40)	1. Are you looking to change a specific behavior (e.g., sedentary lifestyle)? If so, is this a top priority? 2. Have you tried to change this behavior before? 3. Do you believe there are inherent risks/dangers associated with not making this behavioral change? 4. Do you have support for making this change from friends, family, and loved ones?
5. Motivation and decision-making	Understand the reasons and motivations for individuals to practice regular PA in five dimensions: (1) Enjoyment; (2) Competence/Challenge; (3) Fitness; (4) Health; and (5) Social.	Motivation for the participation in physical activities	The Motives for PA Measure - Revised (MPAM-R) (66, 67)	How motivated do you feel to do PA in the following reasons? 1. Because I like engaging in activities which physically challenge me 2. Because I want to maintain my physical health and wellbeing to live a healthy life. 3. Because I feel physically out of shape and want to improve my appearance. 4. Because I want to be physically fit. 5. Because I enjoy spending time with others doing this activity.
	Explore critical thinking, elicit change talk, empower self-transformation, and build SMART goals (Specific, Measurable, Attainable, Relevant, and Time-based) Explore collaborative decision making (Help to solve the paradox of choice dilemma)	Define SMART goals Elicit decision-making	Motivation interviewing script Health economics (gamification, choice architecture, information provision, use of “nudges” to improve decision making)	OARS questions: Open-ended question, Affirmation, Reflective listening, and Summarizing. 1. What does be healthy look like to you? Give me the three best reasons. 2. What is currently motivating you to change? 3. How could you go about moving forward toward your goal? 4. What is holding you back? 5. What do you think would be a good first step?
6. PA Self-regulation	Evaluate the use of measures of self- regulation strategies in the dimensions of planning, scheduling, and self-organizational behaviors	Self-regulation strategies	PA Self-Regulation (PASR-12) (50)	In the last month, how often do you… 1. … set short term goals to stay physically active. 2. … asked a PA expert or health professional for PA advice or demo. 3. … reminded yourself of PA health benefits. 4. … rearranged your schedule to ensure you had time for PA. 5. … purposely planned ways to do PA when on trips away from home.
7. PA Self-efficacy	Assess how sure participants are that they would perform regular PA under different conditions or constraints.	Self-efficacy	PA Self-efficacy Questionnaire (68, 69)	How sure are you to practice PA when… 1. … you are feeling tired. 2. … you are feeling under pressure to get things done. 3. … you are feeling down or depressed. 4. … you have too much work to do at home. 5. … there are other more interesting things to do.
8. PA and exercise preference and tolerance	Assess physical exercise intensity preference, affective responses to exercise, and frequency of vigorous exercise tolerance	Exercise mode and duration preferences Exercise tolerance (intensity)	Preference for and tolerance of the intensity of exercise questionnaire (PRETIE-Q) (70)	What mode of exercise do you prefer? What exercises would you be willing to do? How frequently would you be willing to do PA? For how long? What is your agreement level of the following affirmation? 1. I'd rather go slow during my workout, even if that means taking more time. 2. While exercising, I try to keep going even after I feel tired or exhausted. 3. I'd rather slow down or stop when a workout starts to get too tough. 4. The faster and harder the workout, the more pleasant I feel.
9. PA perception and knowledge	Identify the participants' knowledge about the benefits, limitations and purposes of PA and exercise, consequences of a sedentary lifestyle, and analyze their PA perception	PA knowledge Perceived PA benefits and limitations.	PA knowledge and perception questionnaire (71)	1. Do you consider that you have enough knowledge about PA and exercise? 2. Do you consider regular PA important for a healthy aging? 3. What perception do you have about the benefits of the practice of PA toward your health and the people of your age?

Following, participants were invited to participate in a remote focus group using the *Zoom Video* platform. A subcohort of 16 participants, divided into two groups, participated in the focus groups. The researcher-moderator followed a pre-established script to guide the group discussion (mean duration of 96 min). The focus groups were recorded in audio and video, transcribed in full, and analyzed independently by three researchers using the content analysis proposed by Bardin (72). All participants provided electronic informed consent, and all procedures were approved by the local institutional ethics committee. Detailed methods are provided as Supplementary Material.

## Results

Four major themes and their respective sub-themes emerged from the focus group analysis. We present additional supporting data from the questionnaire's battery, as well as strategies to address the concerns raised, considering the challenges and demands of the clinical setting.

### Age and Aging Are Determinants in Physical Activity Practice

Among all sociodemographic and potential social determinants of health assessed, our participants listed their age and the aging process as major determinants of PA adherence. In general, the expression “at our age/my age” was mentioned by nearly all participants. Age appears as a stimulus to PA, as participants recognize the need to remain healthy and active:

...People at our age need to stay active…a lot of things are needed: physical activity, a good nutrition, occupational therapy, reading, a group of friends. That's what will make us an active person… (P8, 63)

In contrast, for some participants, aging can be a limiting factor for performing regular PA. First, more than 60% of participants “feel tired” or “low on energy” frequently or almost every day. Usually, the shared feelings and symptoms (falls, fatigue, muscle soreness, poor sleep quality, feeling down or depressive) are linked to the sedentarism's consequences. Second, this may be due to the limited perception that older adults build about themselves as fragile, a social burden, and because of cultural issues imposed by society (73):

After we retire, then we take a break, we are a little out of breath...The mind works, but the body doesn't follow along, we have to do everything in a moderate pace, with no rush... (P4, 68)

Aging makes it a little difficult because the gym I attend, there are only me and a gentleman at our age, most of them were young. Then, at your age, I think it demotivate. (P9, 69)

### Physical Activity and Holistic Health: Healthy Mind, Healthy Body

A large proportion of the participants reported moderate perceived stress (92%) and depressive symptoms (40%). Surprisingly, nine out of 10 participants indicated having knowledge that regular PA can help mitigate the symptoms of depression, anxiety, and stress. Participants also recognized that the lack of regular PA can contribute to the development and worsening of depressive symptoms (74), and that directly affects work-related activities, leisure time, household activities, and getting along with people:

Physical activity is good for the human being...it is good for our body, physically as well as mentally. (P12, 81)

Maintaining both an active mind and autonomy was cited as “extremely important.” Surprisingly, however, older adults did not immediately consider planned exercise as part of being “active,” which was instead more often interpreted as maintaining an active mind, having friends, caring for their home, cognitive tasks, etc:

For me, an active older adult is an individual who knows how to solve problems by himself... who has no difficulty participating in tasks and demands [refers to household and handcraft work], on a daily basis... (P13, 75)

### Motivators and Barriers to Physical Activity and Exercise Practice

Although the participants reported that practicing PA was important to them and noted that achieving this goal would represent a challenge for them, it appears that they were unprepared to deal with all aspects of behavior change. Participants mainly focused on the act of starting an exercise program but did not necessarily consider the continued motivation to remain active and develop continued habits of this practice.

With regards to internal stimulus factors, motivation and self-esteem were mentioned as a priority, including the selection of preferred activities targeting health-related issues, such as the prevention or mitigation of diseases and symptoms that affect mental health:

We must have motivation, because when you determine something and focus on what you want, then yes...you have results. (P05, 60)

We have to do what is pleasurable for you, and not just do it for the sake of doing it. What requires you participate in an activity? Enjoyment! (P09, 69)

The external stimulus factor considered most relevant for the participants was related to the social engagement with other older adults, whether it was a group of friends, a partner or family, or the guidance of an exercise professional.

I found a way to do exercise...it's creating a group of friends. One always tries to know how the other is doing and encourages the other to go when he didn't want to, right?…a family that take cares of each other. (P14, 69)

“Being lazy or unmotivated” (65%), and “lack of company” (60%) were the perceived barriers that had the greatest impact on their PA engagement. This finding reveals that older adults fail to invest more effort in an area that they most need to increase their PA levels. Among all five domains (physical, personal, beliefs, motivational, and environmental factors) of perceived barriers to PA, the motivational aspect was the most prominent. Motivation sources were related to maintaining strength and improving physical health and wellbeing. However, despite the desire to improve strength and physical health, 65% of older adults were not motivated to do physically challenging activities. This highlights two interesting findings: (1) lack of broad dissemination of cognitive benefits of being physically active; and (2) a potential opportunity for setting realistic expectations regarding strength and physical improvements (which do require physical challenge).

Participants also demonstrated low self-efficacy and a general lack of self-regulation strategies. Approximately 60%−80% of the participants reported not doing PA or being unsure if they would do it in the following circumstances: “feeling tired or under pressure,” “feeling down,” “too much work at home,” “away from home,” and “no family or friends support.” Additionally, about 70% reported that they never or rarely “rearrange their schedule to ensure they have time to exercise,” or “ask for advice or an exercise demonstration from a health professional expert.” In contrast, 80% of the individuals revealed reminding themselves of exercise health benefits. This indicates that older adults are generally unprepared to deal with the obstacles that are normal and inherent to adopting a lifestyle change (68).

For me, it's laziness, because...we're already tired from work, and still be submitted to walk a lot?! Oh, laziness! [Enthusiastically]. (P10, 65)

…although it is a medical recommendation, it makes me lazy...to do exercise, to follow an exercise routine… (P14, 69)

The most frequent external barriers were financial barriers and those related to the lack of or inadequacy of public places and facilities for the PA practice near home. This highlighted the importance of considering social determinants of health and contextual factors and providing resources that can help to guide individuals through their behavior change journeys (75).

There are many aspects that demotivate people. One aspect is financial. We lack public places to practice physical activities. (P08, 63)

### Guidelines and Professional Recommendations

Clinical PA recommendations are general and did not consider older adults' preferences and values, and physical and environmental limitations (76), and this was cited as a major reason why participants did not tend to follow through with current guidelines or general exercise prescriptions. In our sample, 93% reported that had received general information from health care providers to practice PA. However, participants often mentioned their singularities and identified a lack of consideration of their differences as an obstacle to their potential engagement in PA. Thus, recognizing the singularities in each individual can be a powerful strategy for greater PA engagement. Tailored recommendations, improved knowledge and understanding, and self-perception of fitness and overall health is crucial for the correct implementation and engagement in a PA program. For instance, despite the largest majority (90%) considering regular PA important or a requisite for healthy aging, ~15% had no knowledge and 60% of older adults revealed that would like to learn more about PA and exercise:

*I understand that the person by himself doesn*'*t have…this knowledge to decide on this. I think…the competent authorities…the doctors, all the scientists, the physical therapists…whoever deals with this area is the one who should make these recommendations because all older adults do not have the same vitality...the same physical condition, so that we couldn't...generalize… (P15, 82)*

## A Multidimensional Model of Physical Activity Engagement

Taken together, these findings suggest that supporting lifestyle change especially related to PA requires a stronger partnership between the clinicians and patients/clients. In an ideal scenario, clinicians would learn about exercise recommendations, but also how to perform a detailed investigation of an individual's current stage of behavior change, and be proficient in the use of motivational interviewing concepts to elicit change talk toward behavior change and empower self-transformation. All these components benefit clinicians build tailored physical activity guidelines for each patient/client's functional and cognitive capacity, and maximizing exercise-related effects on motor and brain health (77). However, this may not be feasible in many clinical care settings, due to constraints in time, and the need for specialized training. For this reason, in the current manuscript, we suggest a tool kit including a clinical-meaningful script of questions that can guide all health care providers to collect valuable preliminary data about their patients, and help them to partner and build achievable goals, and provide care within the context of each patient and their family. A similar strategy of a multimodal PA education and coaching intervention was shown to be feasible, well-accepted, and effective in promoting PA participation and cognitive improvement in sedentary older adults with memory complaints (54).

Evidence-based practice highlights the importance of the integration of the knowledge and use of the best evidence, the clinical expertise/expert opinion, and respect for patients' desires and values. For instance, approximately one-third of participants revealed “no intention to change their sedentary behavior in the next 6 months,” two-thirds already failed to change their sedentary behavior, and the half sample did not consider changing behavior a high priority. Despite being screened as sedentary, 37% considered themselves physically active, which demonstrates a limitation on knowledge of exercise and PA concepts and clear recommendations. Thus, the clinician's decision making to investigate these factors and foster building a rapport for a better professional-patient relationship is crucial to elicit change talk and consequently initiate preparation for change.

**Figure 1:**
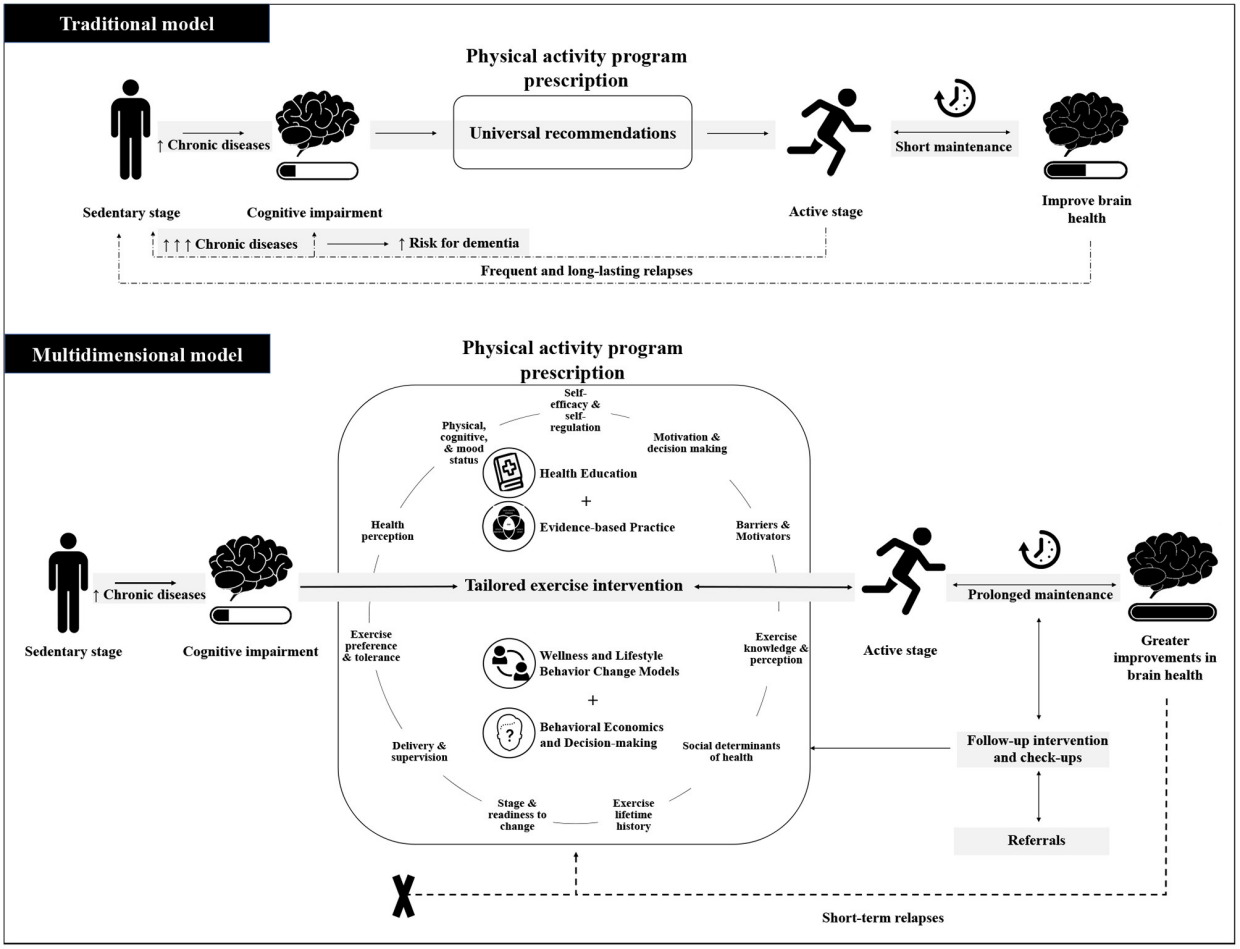
Schematic illustration of traditional model vs. multidimensional model. The traditional model emphasizes universal physical activity recommendations as the main component of an exercise program. The multidimensional model focus on implementing simple ways to effectively translate current guidelines tailored to the needs of the aging individual by screening all listed factors believed to influence adherence and combining all available strategies including health education, evidence-based practice, wellness and lifestyle behavior change models, and behavioral economics and decision-making strategies. The successful assessment and intervention prescription will affect expected prolonged maintenance in a physically active stage and greater improvements in brain health.

We propose three concrete steps to replace the traditional model of exercise recommendation (Figure 1): (1) a multidimensional model of PA engagement, (2) a comprehensive tool kit for clinicians to assess PA adherence-related factors, and (3) a list of items and questions designed to summarize the main components for a quick assessment of PA adherence-related factors (Table 1). Importantly, frequent follow-up assessments and referrals when needed will also be crucial to minimizing relapses.

### Toolkit and a Screening Questionnaire for Clinicians: What Do They Need to Assess?

In Table 1, we propose a list of instruments, questionnaires, and behavior change models that are crucial for clinicians to have a comprehensive understanding and assessment of their patients before prescribing a PA program (Table 1), including specific questions that should be explored by clinicians for a quick screening participant's profile. This screening will also help direct the interview with active listening, build rapport with the patient/client, and gather all necessary information before listing goals and interventions in partnership with the individual.

Future studies will implement the proposed screening targeting older adults at risk for cognitive decline aiming at evaluating validity, reliability, and responsiveness to a multimodal intervention targeting long-term PA participation.

## Conclusion

Our findings suggested four main themes linked to PA adherence in sedentary older adults. First, older adults believe that aging is a limiting factor for PA engagement. Second, maintaining both an active mind and autonomy are priorities, but older adults did not immediately consider planned exercise as part of being “active,” which was instead more often interpreted as maintaining an active mind, having friends, caring for their home, etc. Third, motivational challenges in PA engagement were noted. And fourth, clinical PA recommendations were general and did not consider their physical and contextual limitations, and thus, they do not tend to follow through with them. We presented a multidimensional model of PA adherence to maximize brain health in older adults and suggest a tool kit and key questions to effectively screen sedentary aging adults and translate current guidelines into the needs of the individual by using behavior change strategies.

## Data Availability Statement

The raw data supporting the conclusions of this article will be made available by the authors, without undue reservation.

## Ethics Statement

The studies involving human participants were reviewed and approved by Alagoas State University of Health Science institutional Ethics Committee. The patients/participants provided their written informed consent to participate in this study.

## Author Contributions

DC: study concept, design, data collection, data analysis, data interpretation, and manuscript preparation, writing, editing, and submission. VS: data collection, data processing, data interpretation, and manuscript editing and review. MS, GL, and AM: data collection, data interpretation, and manuscript review. LC and AP-L: critical revision of manuscript for intellectual content. AB: study design, data analysis, and manuscript editing. AO and JG-O: study concept, design, supervisor of study, manuscript writing and editing, and critical revision of manuscript for intellectual content. All authors contributed to the article and approved the submitted version.

## Funding

AO, GL, and AM were supported by the National Council for Scientific and Technological Development and Alagoas State University of Health Science (Brazil).

## Conflict of Interest

JG-O works as Director of Interventional Therapy at Linus Health and AP-L is a co-founder of Linus Health and TI Solutions AG. The remaining authors declare that the research was conducted in the absence of any commercial or financial relationships that could be construed as a potential conflict of interest.

## Publisher's Note

All claims expressed in this article are solely those of the authors and do not necessarily represent those of their affiliated organizations, or those of the publisher, the editors and the reviewers. Any product that may be evaluated in this article, or claim that may be made by its manufacturer, is not guaranteed or endorsed by the publisher.
