# Supplementary material for: Recommending Physical Activity to Your Aging Patients? What Clinicians Need to Know to Increase Adherence From the Older Adult Perspective
Source: Front Rehabil Sci. 2022 Jul 8;3:923221. doi: 10.3389/fresc.2022.923221 (PMC9397884; doi:10.3389/fresc.2022.923221)
Supplement: Supplementary file 1 [file Table_2.DOCX]

Supplementary Material

# Supplementary Methods

## Research Design and Procedures

***Experiment procedures.*** In this perspective study, we support our proposed multidimensional model by collecting data from a mixed-method study: 1) a descriptive-exploratory analysis of a structured electronic questionnaires battery related to exercise engagement, and 2) a qualitative content analysis of the focus group. After the electronic consenting, participants were directed to complete a battery of 11 questionnaires related to demographic characteristics and exercise engagement on the *Qualtrics XM® survey* platform. Following, participants who fully completed the questionnaires were invited to participate in a remote focus group using the *Zoom Video Communications* platform.

***Outcome measures***. The following outcomes were assessed in the structured questionnaire battery: 1) Sociodemographic characteristics and social determinants of health, 2) current knowledge and self-perception of exercise, 3) lifetime physical activity history, 4) exercise preference and tolerance, 5) state of mind and stress, 6) exercise barriers and motivators, 7) stage of behavior change and level of readiness to change, 8) physical activity motivation, 9) physical activity self-efficacy, 10) physical activity self-regulation, and 11) exercise delivery preferences and technology satisfaction. Details of outcomes and questionaries are provided in table 1. The evaluation of these measures was based on the literature review of potential factors influencing physical activity adherence and experts’ opinion. In accordance, valid and reliable measures were selected to compose the assessment battery. References of assessment are provided in the main document. Two additional references of resources for the outcomes are provided below.

***Focus group.*** Focus groups contained a maximum of 10 individuals each. Individuals that agreed to participate in the second part of the study were contacted and invited to participate in the remote focus group on a pre-specified date. Participants received a checklist with the procedures to follow before the group and a team member were available to troubleshoot any issues that would interfere with their full participation. The virtual platform *Zoom Video Communications* were used as an interface for meeting the researchers and the other participants. Audio and video images were recorded. The researcher-moderator followed a pre-established script to guide the group discussion. The estimated duration of the focus group was approximately 120 minutes. Participants were coded by the ordinal sequence they presented themselves, followed by age (e.g., P1, 65). The number of focus groups and individuals selected to participate in the focus groups were determined by the criterion of data saturation, a common method used in qualitative research. This method accounts for the guarantee of sufficient data and a reliable sample. It must consider the use of a sufficient number of interlocutors that encourage recidivism and information complementarity. The focus groups were transcribed in full from the audio and video recordings and analyzed using the content analysis proposed by Bardin. This method consisted of organizing the collected material, successive readings and re-readings, and seeking to apprehend the nuclei of relevance and organization to achieve the objectives proposed for the studied phenomenon. The definition of the thematic categories described in the results of this study was based on the independent analysis of three researchers and disparities were solved in a committee discussion. The results with themes and subthemes represented the agreement among research analyses

## Participant’s recruitment and Eligibility criteria

This study was conducted in collaborative work with the Human Aging Research Group at the Alagoas State University of Health Sciences in Brazil. Participants aged 60 years or older were recruited through the local university community programs for aging adults, brochures and posters placed in common areas of the university, social media, and local basic community health centers. Individuals interested in participating in the study were first formally contacted via telephone and screened for eligibility. Additional inclusion criteria were: 1) sedentary status defined as <75 minutes of vigorous or 150 minutes of moderate activity per week; 2) basic computer skills (accessing an email or using the internet), and 3) free of any other neurological or psychiatric diagnostic and physical limitation that impairs fully participation in the study procedures and exercise program. All participants provided electronic informed consent. The study protocol is approved by the Alagoas State University of Health Sciences institutional review board.

## Additional references

1. Chase J-AD. Interventions to Increase Physical Activity Among Older Adults: A Meta-Analysis. *The Gerontologist*. 2015;55(4): 706–718. https://doi.org/10.1093/geront/gnu090.

2. Rhodes RE, Boudreau P, Josefsson KW, Ivarsson A. Mediators of physical activity behaviour change interventions among adults: a systematic review and meta-analysis. *Health Psychology Review*. 2020; 1–15. https://doi.org/10.1080/17437199.2019.1706614.
